# Digital health technologies for the management of sarcopenia in patients receiving maintenance hemodialysis: a narrative review

**DOI:** 10.3389/fnut.2026.1873406

**Published:** 2026-06-17

**Authors:** Ting Luo, Ping Wei, Huilan Wang, Yuzi Zhao, Yuhang Chen, Rong Zeng, Xueqin Gan, Qian Yang

**Affiliations:** 1 School of Nursing, Chengdu Medical College, Chengdu, China; 2 Jianyang People's Hospital, Chengdu, China; 3 The First Affiliated Hospital of Chengdu Medical College, Chengdu, China

**Keywords:** chronic disease management, clinical validation, digital health technologies, maintenance hemodialysis, narrative review, sarcopenia

## Abstract

Digital health technologies are increasingly being explored as supportive tools for managing sarcopenia in patients undergoing maintenance hemodialysis (MHD). Wearable devices, mobile health applications, telemedicine platforms, artificial intelligence, and virtual or augmented reality may support monitoring, patient engagement, and intervention delivery. However, the evidence remains heterogeneous, and the clinical readiness of different digital modalities varies substantially. Activity monitors, mobile applications, and remote follow-up platforms are relatively feasible for clinical or home-based supportive management, whereas continuous biosensors and some AI-driven prediction systems remain largely at the proof-of-concept or early validation stage. This narrative review synthesizes current applications, potential effects, limitations, and future directions of digital health technologies in the management of sarcopenia among MHD patients. Particular attention is given to internationally representative evidence because many existing studies are concentrated in specific regional or healthcare contexts. The review also discusses how digital tools may support MHD-specific monitoring by integrating data on interdialytic weight gain, volume fluctuations, dialysis adequacy, dialysis- and non-dialysis-day activity patterns, and laboratory indicators related to nutrition, inflammation, and uremic toxin burden. Further multicenter studies, quantitative comparisons across modalities, and rigorous clinical validation are needed before these technologies can be widely implemented.

## Background

According to the Global Kidney Health Atlas published by the International Society of Nephrology (ISN), approximately 850 million people worldwide are living with kidney disease, making chronic kidney disease a major global public health challenge (1). Among patients who progress to kidney failure, maintenance hemodialysis (MHD) remains one of the most widely used kidney replacement therapies, although the number of patients receiving MHD varies substantially across countries, registries, and healthcare systems (1). Although advances in dialysis technology have substantially prolonged survival among patients receiving MHD, sarcopenia—defined as a geriatric syndrome characterized by reduced skeletal muscle mass, decreased muscle strength, and/or impaired physical function—remains highly prevalent in this population, affecting 31.5–42.1% of patients (2, 3). Sarcopenia not only contributes to reduced muscle strength, functional decline, and poorer quality of life in patients undergoing MHD (4) but also significantly increases the risks of complications, such as infection and fracture, with mortality risk rising by two- to threefold (5). Consequently, it imposes a substantial burden on both families and healthcare systems.

The pathogenesis of sarcopenia in MHD patients is complex and distinct, involving the interplay of multiple factors, such as chronic microinflammation, uremic toxin accumulation, metabolic acidosis, protein-energy wasting, dialysis-related amino acid loss, and physical inactivity (6, 7). Conventional management strategies mainly include nutritional supplementation, pharmacologic therapy, and routine exercise guidance; however, several persistent barriers limit their effectiveness in the MHD population: (1) inadequate self-management capacity and poor adherence; (2) lack of individualized interventions tailored to different stages of disease and functional status; and (3) insufficient follow-up and monitoring, making it difficult to adjust intervention intensity dynamically (8). These limitations contribute to the suboptimal clinical effectiveness of traditional management approaches for sarcopenia in patients receiving MHD.

Digital health technologies have created new opportunities to address these challenges. The World Health Organization defines digital health technologies as digital tools—such as wearable devices, mobile health applications, telemedicine, and artificial intelligence—used to support health management, disease prevention, diagnosis, and treatment (9). These technologies have shown value in chronic disease management, such as cardiovascular disease and diabetes, and their application in MHD-related sarcopenia is gradually emerging (10, 11).

Nevertheless, several important gaps remain in the current literature. First, there is an evidence gap: most intervention studies have focused on the general older adult population and have not adequately accounted for the unique characteristics of MHD patients, such as fluctuations in volume status, differences in physical activity between dialysis and non-dialysis days, and the effects of uremic toxins on muscle metabolism. In addition, high-quality randomized controlled trials and long-term follow-up data remain limited. Second, there is a technology gap: the lack of standardized data formats across digital platforms contributes to fragmented information systems, while the clinical validation and interpretability of artificial intelligence algorithms remain insufficient. Third, there is an implementation gap: digital literacy, willingness to adopt technology, and concerns regarding privacy and data security, particularly among older patients and those with lower educational attainment, have not been systematically evaluated.

Given these considerations, this review emphasizes three issues that are particularly important for digital management of sarcopenia in MHD. First, digital health technologies should be integrated with MHD-specific indicators, such as interdialytic weight gain, volume overload, blood pressure variability, ultrafiltration volume, dialysis adequacy, inflammatory status, nutritional biomarkers, and laboratory indicators associated with uremic toxin burden. Combining these data with activity, muscle strength, diet, and patient-reported symptoms may help clinicians distinguish true functional decline from dialysis-related fluctuations (12). Second, evidence from different countries, healthcare systems, and technological environments should be considered to avoid overgeneralizing from a single regional context (11). Third, clinically available tools should be distinguished from validation-stage and conceptual technologies to avoid overstating the readiness of continuous biosensors, AI-based models, and virtual rehabilitation systems.

In light of these gaps, this narrative review aims to provide a structured synthesis of the forms, potential applications, intervention effects, limitations, and future directions of digital health technologies in the management of sarcopenia among patients undergoing MHD, thereby offering a balanced reference for their clinical translation and standardized application.

## Methods

### Literature identification and evidence synthesis

This article was conducted as a narrative review rather than a systematic review or scoping review. Its purpose was to provide a structured synthesis of current evidence, conceptual developments, clinical applications, and implementation challenges related to digital health technologies for managing sarcopenia in patients undergoing MHD. As the available literature is heterogeneous and includes clinical studies, systematic reviews, technology-focused studies, conceptual papers, policy documents, and pilot or feasibility studies, a narrative approach was considered appropriate for integrating evidence from multiple disciplinary perspectives.

A literature search was conducted in PubMed/MEDLINE, Web of Science, Embase, Cochrane Library, Scopus, CNKI, and WanFang databases. The search covered publications from database inception to April 2026. The following search terms were used alone or in combination: “maintenance hemodialysis,” “hemodialysis,” “dialysis,” “chronic kidney disease,” “end-stage kidney disease,” “sarcopenia,” “muscle wasting,” “frailty,” “digital health,” “mHealth,” “mobile health,” “telemedicine,” “telehealth,” “wearable device,” “activity tracker,” “artificial intelligence,” “machine learning,” “virtual reality,” “augmented reality,” “remote monitoring,” “digital literacy,” “uremic toxin,” “protein-energy wasting,” “dialysis adequacy,” and “intradialytic exercise.” Equivalent Chinese search terms were also used in CNKI and WanFang to identify relevant Chinese-language studies.

Studies and documents were considered eligible if they met one or more of the following criteria: (1) focused on sarcopenia, frailty, muscle wasting, physical function, nutrition, exercise, or rehabilitation in patients undergoing MHD; (2) evaluated or discussed digital health technologiessuch as wearable devices, mobile applications, telemedicine platforms, artificial intelligence, virtual or augmented reality, biosensors, or remote monitoring systems; (3) reported dialysis-specific factors relevant to sarcopenia, such as dialysis adequacy, interdialytic weight gain, volume status, inflammation, metabolic acidosis, protein-energy wasting, or uremic toxin burden; or (4) provided methodological, ethical, implementation, or policy perspectives relevant to digital health technologies in chronic disease or kidney care. English- and Chinese-language publications were included. Articles were excluded if they were unrelated to digital health, sarcopenia, dialysis, chronic kidney disease, rehabilitation, or nutritional management; if they were duplicate publications; if the full text was unavailable; or if they were purely commercial or promotional materials without scientific content.

As direct evidence on digital health technologies specifically for MHD-associated sarcopenia remains limited, evidence from related populations was also considered when relevant. These included studies involving patients with chronic kidney disease, end-stage kidney disease, older adults with sarcopenia, frail populations, and broader chronic disease cohorts. However, evidence from non-MHD populations was treated as indirect or methodological support and was not interpreted as direct evidence for MHD-associated sarcopenia. Particular attention was given to whether each study directly involved MHD patients, whether sarcopenia-specific outcomes were assessed, and whether the digital intervention had been clinically validated.

The evidence was synthesized narratively according to technology type and clinical function. Digital health technologies were grouped into wearable devices, artificial intelligence, mobile applications and telemedicine platforms, VR/AR-based rehabilitation, and conceptual or proof-of-concept biosensors. The clinical relevance of each modality was evaluated according to several dimensions: study population, study design, sample size when available, directness of evidence to MHD-associated sarcopenia, reported outcomes, technological maturity, clinical applicability, and major limitations. As the included literature was highly heterogeneous in population, intervention type, follow-up duration, comparator group, and outcome measures, no meta-analysis or pooled quantitative synthesis was performed.

To strengthen evidence appraisal, the included evidence was classified into three broad levels of clinical readiness: clinically available tools, tools under clinical validation, and conceptual or proof-of-concept technologies. Clinically available tools included wearable activity monitors, mobile applications, telemedicine platforms, remote follow-up systems, and digital body-weight or blood pressure monitoring systems. Tools under clinical validation included AI-based prediction models, machine-learning-assisted gait analysis, AI-assisted musculoskeletal ultrasound, and VR/AR-based rehabilitation. Conceptual or proof-of-concept technologies included continuous protein sensors, uremic-toxin-related biosensors, real-time amino acid monitoring systems, and experimental metabolic biosensors. This classification was used to avoid overinterpreting early-stage technologies as established clinical tools.

This narrative review was not registered, and a formal PRISMA flow diagram was not generated because the objective was not to conduct a systematic review or meta-analysis. Nevertheless, the reporting of the literature identification process, evidence selection, and narrative synthesis was guided by relevant principles of transparency for review articles. The limitations of this approach are acknowledged in the discussion, such as the possibility of selection bias, heterogeneity of available evidence, and limited direct evidence specific to digital health technologies for MHD-associated sarcopenia.

### Critical appraisal of representative evidence

As the available evidence was heterogeneous and included different study designs, populations, digital modalities, and outcome measures, a formal quantitative quality score was not assigned. Instead, representative evidence was critically appraised according to study design, population relevance, directness to MHD-associated sarcopenia, reported outcomes, methodological limitations, and clinical applicability. Evidence directly involving MHD patients with sarcopenia was considered more clinically relevant than evidence derived from general older adults, frailty populations, non-dialysis CKD patients, or broader chronic disease cohorts. Evidence from non-MHD populations was used only as indirect or methodological support and was not interpreted as direct evidence of effectiveness in MHD-associated sarcopenia, as shown in Table 1.

**Table 1:** Critical appraisal of representative evidence on digital health technologies for MHD-associated sarcopenia.

Evidence area/representative studies	Population	Study design/evidence type	Directness to MHD-associated sarcopenia	Main findings relevant to this review	Key methodological limitations	Clinical applicability
Wearable activity monitoring and physical activity assessment (21, 23)	MHD patients	Cross-sectional or prospective observational studies using physical activity or wearable-derived data	Moderate to high; studies involve MHD patients, but not all focus specifically on sarcopenia	Wearable or activity data may help characterize physical activity patterns, dialysis-day differences, sedentary behavior, and potential functional decline	Mostly observational; limited ability to infer causality; sarcopenia-specific outcomes, such as muscle mass or handgrip strength are not always assessed	Clinically feasible for supportive monitoring, but direct evidence for improving sarcopenia outcomes remains limited
Wearable gait sensors combined with machine learning (22)	Frailty or broader older/chronic disease populations	Digital gait assessment with machine-learning prediction	Indirect; frailty is related to sarcopenia, but the population is not specific to MHD-associated sarcopenia	Provides a methodological reference for using gait parameters and machine learning to identify functional risk	Limited dialysis-specific variables; external validity to MHD patients is uncertain; clinical outcome prediction remains insufficient	Promising for future MHD-specific validation, but not yet a mature diagnostic tool
Sarcopenia screening tools and digital screening support (45)	MHD patients	Comparative diagnostic study of sarcopenia screening tools	High for screening in MHD patients, but not necessarily digital-health-specific	Validated screening tools such as SARC-CalF may be integrated into digital platforms to improve screening efficiency	Screening tools still require confirmation by standard sarcopenia assessment; digital implementation itself was not the main intervention	Useful for app-based or platform-based screening, but should not replace clinical assessment
AI-based sarcopenia prediction models (29)	General chronic disease population	Machine-learning prediction model and web-based tool	Indirect; model not developed specifically in MHD patients	Demonstrates feasibility of AI-based sarcopenia risk prediction and web-based decision support	Population mismatch; lack of dialysis-specific variables; external validation in MHD populations is lacking	Methodologically informative, but direct clinical use in MHD-associated sarcopenia is premature
MHD prognostic prediction model (34)	MHD patients	Clinical prediction model for mortality and survival	Moderate; population is MHD, but outcome is mortality rather than sarcopenia	Shows that implementable prediction modeling is feasible in dialysis populations	Not designed for sarcopenia prediction; does not directly evaluate muscle function or digital intervention effects	Useful methodological reference for future MHD-specific sarcopenia prediction models
AI-assisted musculoskeletal ultrasound and sarcopenia assessment (46)	MHD patients	Sarcopenia assessment using musculoskeletal ultrasound	Moderate to high; population is MHD and outcome relates to sarcopenia assessment	Gastrocnemius muscle thickness is associated with handgrip strength and skeletal muscle mass index; ultrasound may support objective assessment	AI-assisted interpretation remains mostly conceptual; need multicenter validation and comparison with standard diagnostic criteria	Potentially useful for standardized assessment, but AI-assisted ultrasound remains under validation
Mobile health and telemedicine in chronic dialysis or CKD (36–38)	CKD, ESRD, chronic dialysis, or mixed kidney disease populations	Scoping review, systematic review, app evaluation, or implementation studies	Moderate; kidney disease relevant, but sarcopenia-specific outcomes are limited	mHealth and telemedicine may support self-management, dietary tracking, remote monitoring, education, and communication	Heterogeneous interventions; variable study quality; limited direct assessment of muscle mass, handgrip strength, or physical performance	Clinically feasible as supportive care tools, but their incremental effect on sarcopenia outcomes remains uncertain
COVID-19-related telemedicine and remote care experience (39)	Patients and staff using telehealth services	Qualitative or implementation experience	Indirect; supports telehealth feasibility but not specific to MHD sarcopenia	Provides evidence that telehealth may maintain care continuity and reduce unnecessary visits	Often not dialysis-specific or sarcopenia-specific; pandemic context may limit generalizability	Useful for implementation planning, but not direct evidence of sarcopenia benefit
Exercise interventions in MHD-associated sarcopenia (31–33)	MHD patients with sarcopenia or related functional impairment	Meta-analysis, network meta-analysis, or intervention study	High for exercise effectiveness; indirect for digital delivery	Resistance exercise, multicomponent exercise, and intradialytic exercise may improve handgrip strength, 6-min walk distance, muscle mass, gait speed, or nutritional indicators	Demonstrates benefit of exercise itself, not necessarily the added value of digital platforms; intervention protocols vary	Supports the clinical importance of exercise; digital tools may help deliver and monitor exercise, but added value requires comparative trials
VR-based rehabilitation experience (41)	MHD patients	Qualitative study of VR rehabilitation experience	Moderate; population is MHD, but evidence is qualitative and not sarcopenia-specific enough	VR may improve exercise motivation, engagement, and emotional experience	Small or qualitative evidence; lacks randomized comparison; objective functional outcomes and long-term effects remain unclear	Promising for engagement, but still investigational for improving sarcopenia outcomes
VR safety and usability evidence (42–44)	Hemodialysis, older adults, or rehabilitation populations	Systematic review, feasibility, or usability studies	Indirect to moderate; relevant to safety and implementation rather than direct sarcopenia efficacy	Highlights risks such as intradialytic hypotension, visual fatigue, dizziness, motion sickness, falls, cost, and usability barriers	Evidence often from non-MHD or non-sarcopenia populations; limited long-term safety data	Important for implementation planning, but clinical effectiveness remains unproven
Digital health literacy and technology acceptance (55–59, 66–69)	Older adults, diverse socioeconomic groups, CKD/MHD, or general populations	Panel studies, qualitative studies, literacy assessment studies, reviews	Indirect to moderate; relevant to implementation rather than sarcopenia efficacy	Digital literacy, socioeconomic status, age, cognitive function, language, and infrastructure affect adoption and adherence	Often not MHD-specific; variable measurement of digital literacy and technology acceptance	Highly relevant for equitable implementation, especially in older or disadvantaged MHD patients
Conceptual biosensors, such as continuous protein sensors (20, 64)	Conceptual, non-MHD, or early-stage technology contexts	Proof-of-concept or technology-focused evidence	Low directness; not clinically validated in MHD-associated sarcopenia	May inspire future monitoring of protein intake, amino acid loss, metabolism, or uremic toxin burden	No validated MHD sarcopenia outcomes; technical reliability, safety, and cost-effectiveness unknown	Should be discussed only as future research direction, not as current clinical tool

This critical appraisal indicates that the strongest direct evidence in MHD-associated sarcopenia currently comes from studies of sarcopenia screening, exercise intervention, and selected rehabilitation or functional assessment approaches. However, these studies do not necessarily demonstrate that digital technologies themselves provide additional benefit beyond conventional care. Evidence for wearable devices, mobile applications, and telemedicine is more mature in terms of feasibility and supportive monitoring, but remains limited regarding direct sarcopenia outcomes. Evidence for AI models, AI-assisted ultrasound, VR/AR rehabilitation, and biosensors is promising but less clinically mature, with many studies relying on indirect populations, pilot designs, qualitative findings, or proof-of-concept data. Therefore, the findings of this narrative review should be interpreted according to the directness, methodological quality, and clinical readiness of each evidence category.

## Conceptual framework and core features of digital health technologies

Digital health technologies refer to an integrated system of digital tools and platforms, such as, but not limited to, wearable devices, mobile health applications, telemedicine systems, artificial intelligence, and virtual reality, designed to support health management, disease prevention, diagnosis, treatment, and rehabilitation. In its Global Strategy on Digital Health 2020–2025, the World Health Organization (WHO) identifies digital health as a key enabler of universal health coverage and the Sustainable Development Goals (13). The core features of digital health technologies can be summarized into three dimensions: (1) real-time data collection, whereby physiological, behavioral, and environmental data are acquired through sensors or active user input; (2) intelligent analysis, in which algorithm-driven models are used for risk identification, trend prediction, and personalized recommendations; and (3) dynamic feedback, through which visualized reports, alerts, or intervention suggestions are delivered to users or healthcare providers (14, 15).

In the field of chronic disease management, digital health technologies have evolved from simple monitoring tools into integrated management platforms encompassing the full continuum of care, such as disease surveillance, risk stratification, individualized intervention, and continuity of care (16). Their value is particularly relevant to the management of sarcopenia among patients undergoing MHD. These patients require long-term and continuous monitoring of muscle mass, muscle strength, physical activity, nutritional intake, and functional status, yet traditional care models often fail to support frequent, individualized, and dynamically adjusted interventions. The real-time and interactive capabilities of digital health technologies are therefore well-suited to address these limitations (11, 15).

In addition, the management of sarcopenia in MHD patients must extend beyond muscle mass alone and account for dialysis-related and metabolic factors, such as volume status, interdialytic weight gain, ultrafiltration burden, inflammatory status, dialysis adequacy, nutritional impairment, and uremic toxin accumulation. These factors may influence both the development of sarcopenia and the interpretation of body composition or physical function measurements. Therefore, digital health technologies should ideally integrate multisource data, such as wearable-derived activity data, patient-reported symptoms, dietary records, dialysis-related indicators, and biochemical markers. Such multimodal data fusion may help clinicians identify functional decline more accurately, distinguish true sarcopenic progression from dialysis-related physiological fluctuations, and adjust exercise or nutritional interventions in a more individualized manner (17, 18).

### Evidence maturity and clinical readiness of digital health technologies

Although digital health technologies share common features such as real-time monitoring, intelligent analysis, and feedback, their levels of evidence maturity and clinical readiness differ substantially. To avoid overgeneralizing preliminary findings, this review distinguishes digital health technologies into three categories: clinically available tools, tools under clinical validation, and conceptual or proof-of-concept technologies. This classification is particularly important in the field of MHD-associated sarcopenia, where some technologies are already feasible for supportive monitoring, whereas others remain exploratory and require further validation before clinical implementation, as shown in Table 2.

**Table 2:** Evidence maturity and clinical readiness of digital health technologies for sarcopenia management in MHD patients.

Category	Examples	Current interpretation in MHD-associated sarcopenia
Clinically available tools	Activity trackers, accelerometers, pedometers, smart watches, mobile health applications, telemedicine platforms, remote follow-up systems, body weight and blood pressure monitoring systems	These tools are already feasible for supportive management, such as physical activity tracking, symptom recording, exercise supervision, dietary logging, remote education, and follow-up. However, their direct effectiveness on MHD-specific sarcopenia outcomes, such as skeletal muscle mass, handgrip strength, gait speed, hospitalization, and mortality, remains insufficiently quantified.
Tools under clinical validation	AI-based prediction models, machine-learning-assisted gait analysis, AI-assisted musculoskeletal ultrasound, virtual reality rehabilitation, augmented reality-assisted exercise training	These technologies have potential value for risk prediction, automated assessment, personalized intervention, and rehabilitation adherence. However, most available evidence comes from pilot studies, general older adult populations, chronic disease populations, or non-MHD settings. Multicenter validation in MHD patients is required before routine clinical use.
Conceptual or proof-of-concept technologies	Continuous protein sensors, uremic-toxin-related biosensors, real-time amino acid loss monitoring systems, experimental metabolic biosensors	These technologies may provide future opportunities for precision monitoring of nutrition, metabolism, and toxin burden, but they should not be presented as mature clinical tools. At present, they remain conceptual or experimental and require technical validation, clinical validation, safety evaluation, and cost-effectiveness assessment.

This evidence maturity framework helps clarify the role of different digital modalities in clinical decision-making. Clinically available tools may be used to support monitoring and communication, but they should not replace standard sarcopenia assessment methods such as handgrip strength, gait speed, muscle mass evaluation, or clinician judgment. Tools under clinical validation may provide additional predictive or rehabilitative value, but their effectiveness and safety need to be confirmed in MHD-specific populations. Conceptual technologies, such as continuous biosensors, should be discussed as future research directions rather than as currently available clinical solutions (19).

By establishing this distinction, the present review provides a more balanced basis for evaluating digital health technologies in MHD-associated sarcopenia. It also helps avoid overinterpretation of early-stage technologies and supports a clearer transition from technical feasibility to clinical validation and, ultimately, patient-centered implementation.

**Figure 1:**
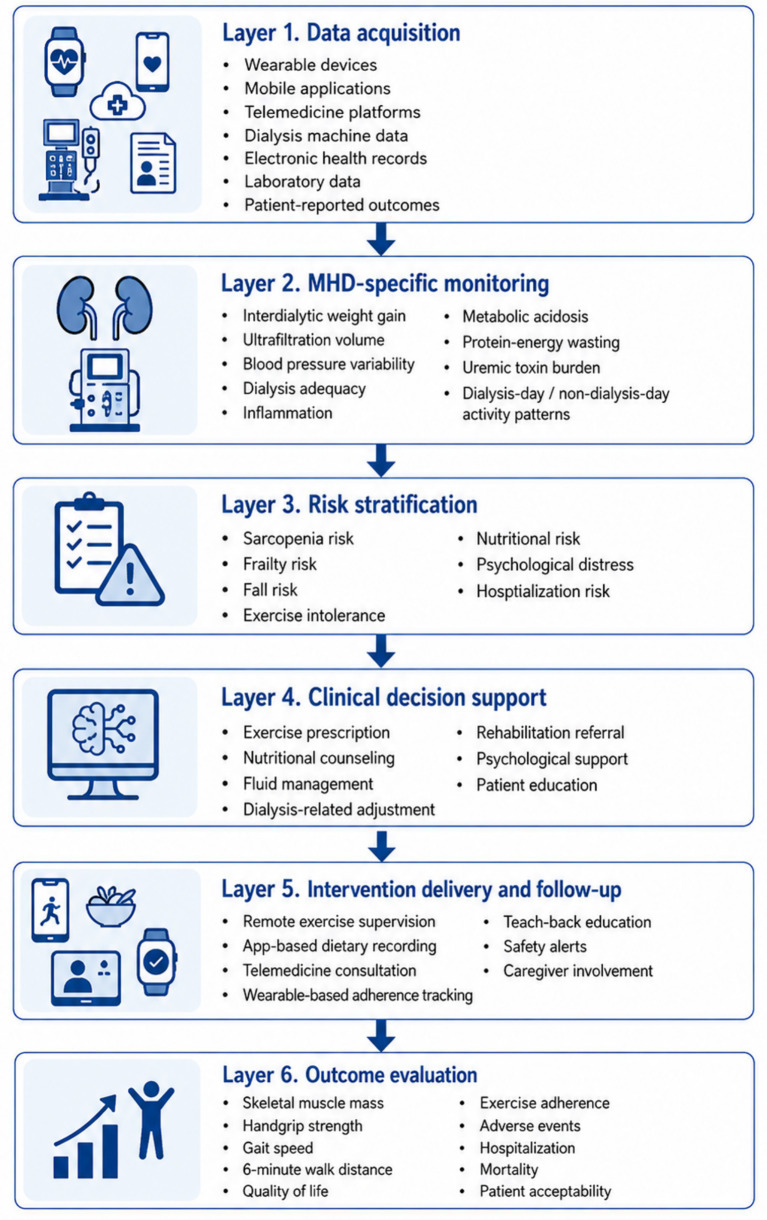
Proposed MHD-specific digital management framework for sarcopenia in patients undergoing maintenance hemodialysis.

To further provide an integrative perspective on clinical implementation, this review proposes an MHD-specific digital management framework for sarcopenia. As shown in Figure 1, digital health technologies should not be applied as isolated tools. Instead, they should be integrated into a multilayer clinical framework that links data acquisition, dialysis-specific monitoring, risk stratification, clinical decision support, intervention delivery, follow-up, and outcome evaluation. This framework may help clarify how different digital modalities can be combined to support patient-centered and clinically meaningful management of sarcopenia in MHD patients.

This framework illustrates how digital health technologies may be integrated into the management of MHD-associated sarcopenia. Data can be collected from wearable devices, mobile applications, telemedicine platforms, dialysis machines, electronic health records, laboratory tests, and patient-reported outcomes. These data should then be interpreted in relation to MHD-specific factors, such as interdialytic weight gain, ultrafiltration volume, blood pressure variability, dialysis adequacy, inflammation, metabolic acidosis, protein-energy wasting, uremic toxin burden, and activity differences between dialysis and non-dialysis days. Through risk stratification and clinical decision support, digital systems may help guide exercise prescription, nutritional counseling, fluid management, rehabilitation referral, psychological support, and patient education. The effectiveness of this framework should be evaluated using clinically meaningful outcomes, such as skeletal muscle mass, handgrip strength, gait speed, 6-min walk distance, quality of life, adherence, adverse events, hospitalization, mortality, and patient acceptability.

## Applications of digital health technologies in the management of sarcopenia in patients undergoing maintenance hemodialysis

### Smart wearable devices

Wearable sensors and biosensors represent one of the most intuitive and practical applications of digital health technologies in the management of sarcopenia (20). However, their clinical readiness varies substantially, and different types of technologies should not be discussed at the same evidence level. In the context of MHD, these technologies can be broadly divided into three categories: clinically available wearable devices, dialysis-related monitoring tools, and conceptual biosensors. Clinically available wearable devices include activity trackers, accelerometers, pedometers, smart watches, heart-rate monitors, sleep monitors, and wearable gait sensors. These devices can continuously collect data on step count, sedentary time, activity intensity, exercise duration, heart rate, sleep patterns, and gait-related parameters, thereby providing objective information for monitoring physical activity and functional decline.

For MHD patients, wearable monitoring has particular relevance because physical activity, exercise tolerance, fatigue, and functional performance may differ between dialysis and non-dialysis days. Hu et al. (21) found that insufficient physical activity is highly prevalent among MHD patients, and that physical activity levels and regular exercise are positively associated with health-related quality of life. These findings support the value of wearable devices as supportive tools for daily activity monitoring and exercise adherence assessment. Nevertheless, currently available wearable devices should be regarded as auxiliary monitoring tools rather than independent diagnostic instruments for sarcopenia. Their major role lies in longitudinal tracking, early identification of reduced activity, and assistance in the individualized adjustment of exercise interventions.

Wearable gait sensors combined with machine learning represent a promising but still validation-stage technology. Fan et al. (22) reported that digital health technology integrating wearable gait sensors and machine learning improved the accuracy of frailty prediction. As sarcopenia is closely related to frailty and is one of its major components (4), this approach may provide a useful methodological reference for the early identification of sarcopenia risk in MHD patients. However, this evidence was derived from a broader frailty-related context rather than from MHD-associated sarcopenia specifically. Therefore, wearable gait analysis, combined with machine learning, should currently be interpreted as a technology under clinical validation rather than a mature diagnostic tool for sarcopenia in MHD patients. Future studies should determine whether wearable-derived indicators, such as gait speed, gait variability, step count, sedentary time, and activity intensity, can predict changes in handgrip strength, skeletal muscle mass index, hospitalization, falls, and mortality in MHD populations (23).

In addition to general activity monitoring, digital tools should be connected with dialysis-related monitoring systems to better capture MHD-specific factors that influence sarcopenia assessment and intervention. Dialysis-related monitoring tools may include electronic body-weight records, pre- and post-dialysis blood pressure monitoring, interdialytic weight gain monitoring, ultrafiltration volume records, bioelectrical impedance analysis, and dialysis adequacy indicators such as Kt/V. These data are important because volume overload, rapid ultrafiltration, intradialytic hypotension, and post-dialysis fatigue may influence muscle performance, exercise tolerance, and body composition assessment (12, 24). Therefore, digital platforms should ideally integrate wearable-derived activity data with dialysis-related variables, such as pre- and post-dialysis weight changes, interdialytic weight gain, blood pressure variability, ultrafiltration volume, dialysis adequacy, and bioelectrical impedance-based volume status.

It should also be clarified that digital tools cannot directly replace laboratory testing for uremic toxins or biochemical abnormalities. However, they may help generate risk alerts by integrating laboratory indicators with behavioral and dialysis-related data. Relevant laboratory indicators may include serum albumin, phosphorus, blood urea nitrogen, creatinine, C-reactive protein, inflammatory markers, and other biochemical indicators associated with nutritional status, inflammation, dialysis adequacy, and uremic toxin burden. When these laboratory data are combined with wearable-derived information, such as step count, sedentary time, sleep status, activity intensity, and exercise tolerance on dialysis and non-dialysis days, digital platforms may help clinicians identify patients at higher risk of sarcopenia progression and adjust nutritional or exercise interventions more precisely (25, 26).

By contrast, conceptual biosensors should be clearly distinguished from clinically available wearable devices. Gottlieb et al. (20) proposed a continuous protein sensor for sarcopenia management in patients receiving glucagon-like peptide-1 receptor agonist therapy. Although this concept may inspire future nutritional monitoring strategies, it has not been clinically validated in MHD patients or in MHD-associated sarcopenia. Therefore, the continuous protein sensor remains a proof-of-concept technology and should be interpreted as a future direction rather than a clinically validated tool for MHD-associated sarcopenia management. Similarly, future biosensors for real-time monitoring of uremic toxins, amino acid loss, protein intake, or metabolic status remain experimental and should not be presented as tools currently available for routine clinical use.

Overall, clinically available wearable devices may support physical activity monitoring, exercise adherence assessment, and early identification of functional decline in MHD patients. Dialysis-related monitoring tools can further provide important contextual information on volume status, ultrafiltration burden, blood pressure fluctuation, and dialysis adequacy. In contrast, continuous protein sensors and uremic-toxin-related biosensors should be classified as conceptual or proof-of-concept technologies. Future research should focus on integrating wearable, dialysis-related, laboratory, and patient-reported data into MHD-specific digital monitoring frameworks and validating their effectiveness in improving sarcopenia-related clinical outcomes.

### Artificial intelligence

Artificial intelligence (AI) encompasses multiple domains, such as machine learning, deep learning, intelligent robotics, natural language processing, and clinical decision-support algorithms. Owing to its capacity to process large volumes of complex and multidimensional data, AI may provide technical support for risk assessment, diagnosis, and individualized management of sarcopenia in patients undergoing MHD (27, 28). However, most AI models discussed in this field are not yet clinically validated for sarcopenia prediction in MHD populations. Existing models have mainly been developed in general older adults, chronic disease populations, or non-dialysis settings. Therefore, AI should currently be regarded as a promising decision-support approach rather than a clinically established tool for routine sarcopenia management in MHD patients. Rong et al. (29) developed a multilayer perceptron-based machine learning model to predict sarcopenia risk in patients with chronic diseases and further created an online application tool. The model identified body weight, age, body mass index, height, total cholesterol, and sex as key predictive features. This study provides a useful methodological reference for AI-based sarcopenia risk prediction. Nevertheless, because the model was developed in a general chronic disease population rather than in MHD patients, its direct applicability to MHD-associated sarcopenia remains limited. In MHD populations, sarcopenia is influenced not only by age, body composition, and chronic disease status but also by dialysis-related uremic factors. Therefore, future AI models should incorporate MHD-specific variables, such as dialysis vintage, dialysis adequacy, ultrafiltration volume, interdialytic weight gain, residual kidney function, serum albumin, C-reactive protein, interleukin-6, and other nutritional and inflammatory indicators. In addition, biochemical indicators related to uremic toxin burden, such as urea, phosphorus, indoxyl sulfate, and p-cresyl sulfate, should be considered when available. Differences in physical activity patterns between dialysis and non-dialysis days may also provide important predictive information for sarcopenia risk stratification.

AI may also support individualized exercise and nutritional management. Liu et al. (30) emphasized that future research should prioritize multimodal therapies, validated biomarkers, and AI-driven algorithms to achieve personalized management of sarcopenia in older adults, particularly in complex populations. This perspective is relevant to MHD patients because their exercise tolerance and nutritional needs are affected by dialysis schedules, fatigue, inflammation, protein-energy wasting, and metabolic disturbances. In theory, AI algorithms could integrate wearable activity data, dietary records, handgrip strength, gait speed, muscle mass assessment, biochemical indicators, and dialysis-related parameters to generate individualized recommendations for exercise intensity, training frequency, protein intake, and follow-up intervals. However, this application remains largely theoretical in the field of MHD-associated sarcopenia and should not be interpreted as established clinical practice.

Evidence from exercise studies supports the importance of rehabilitation interventions in this population. Li et al. (31) and Li et al. (32) reported that resistance training and multicomponent exercise can improve outcomes, such as muscle strength, physical function, muscle mass, and nutritional status in MHD patients with sarcopenia. These findings suggest that individualized exercise intervention is clinically meaningful. However, they do not directly demonstrate that AI-guided exercise prescriptions are superior to conventional clinician-guided interventions. Therefore, AI-driven recommendations for resistance exercise, aerobic exercise, nutritional supplementation, or combined intervention strategies should be described as a future direction requiring prospective clinical validation rather than as a proven management approach. Chen et al. (33) discussed the potential role of AI in risk management for older adults with chronic diseases and sarcopenia, highlighting its value in early diagnosis, personalized treatment, and continuous monitoring. Although these functions are conceptually applicable to MHD patients, the external validity of such models remains uncertain because MHD patients have unique physiological characteristics, such as volume fluctuation, dialysis-related amino acid loss, uremic toxin accumulation, and inflammation-related muscle catabolism. Similarly, Goldstein et al. (34) developed a clinically implementable prediction model for near-term mortality and long-term survival in patients undergoing MHD. Although this model was not specifically designed for sarcopenia, it demonstrates the feasibility of predictive modeling in dialysis populations. Its methodological approach may inform future sarcopenia-related models for predicting incident sarcopenia, frailty progression, falls, hospitalization, quality-of-life decline, and mortality in MHD patients.

Before routine clinical adoption, AI-based sarcopenia models require external validation, calibration across different dialysis populations, and transparent reporting of algorithmic performance (35). Future studies should also evaluate model interpretability, clinical usefulness, fairness across age, sex, socioeconomic status, and ethnic groups, and integration into real-world dialysis workflows. In addition, AI models should be tested in multicenter and internationally representative MHD cohorts to determine whether their predictive performance is stable across different healthcare systems and dialysis practices.

Overall, AI has potential value in risk stratification, individualized intervention, and prognostic assessment for MHD-associated sarcopenia. However, the current evidence remains insufficient for routine clinical implementation. Future research should move beyond general chronic disease or older adult models and develop MHD-specific, externally validated, interpretable, and clinically integrated AI tools. Only after rigorous validation can AI be considered a reliable component of digital health management for sarcopenia in patients undergoing MHD.

### Mobile applications and telemedicine platforms

Mobile health applications and telemedicine platforms provide patients undergoing MHD with tools for self-management, remote follow-up, health education, exercise supervision, dietary recording, and communication with healthcare professionals. Compared with AI-based prediction systems or conceptual biosensors, these platforms are more clinically feasible and have already been used in chronic disease management and kidney care. In the MHD setting, they can help patients record dietary intake, fluid intake, body weight, blood pressure, exercise completion, fatigue, and other health-related data while also delivering education, reminders, and follow-up support (36–38).

International evidence also suggests that mobile health and telemedicine approaches have gradually been applied in kidney disease and dialysis care. Studies from different healthcare systems have explored remote monitoring, mobile applications, patient portals, dietary tracking tools, and telehealth visits for patients with chronic kidney disease or dialysis-dependent kidney failure (36, 37). For example, mobile health interventions in end-stage renal disease have included remote monitoring systems, apps, websites, mobile phones, tablets, and social platforms, with monitored indicators, such as body weight, blood pressure, ultrafiltration, urine volume, dialysis adequacy, albumin, phosphorus, serum creatinine, and blood urea nitrogen. These functions are relevant to MHD-associated sarcopenia because they can support long-term monitoring of nutrition, volume status, dialysis-related burden, and physical function.

Mobile applications may also support dietary and nutritional management, which is central to sarcopenia prevention in MHD patients. Digital food diaries, nutrient calculators, and app-based dietary education can help patients monitor protein intake, energy intake, sodium, potassium, phosphorus, and fluid restriction (38). These functions may be particularly useful for MHD patients, who need to balance adequate protein-energy intake against fluid and electrolyte control. However, it should be emphasized that mobile applications cannot replace individualized assessment by nephrologists, nurses, or dietitians. Their role is mainly to support self-recording, feedback, and communication between patients and healthcare professionals.

Telemedicine platforms may further facilitate exercise-based management of sarcopenia in MHD patients. As patients undergoing MHD often have limited exercise capacity and require professional supervision to ensure that physical activity is both safe and effective, remote platforms may help deliver exercise videos, individualized training plans, symptom check-ins, and progress feedback. The meta-analysis by Li et al. (31) highlighted the effectiveness of resistance training and multicomponent exercise in MHD patients with sarcopenia, suggesting that structured exercise interventions are clinically meaningful in this population. However, the available evidence mainly supports the effectiveness of exercise itself, rather than proving that app-based or telemedicine-based delivery is superior to conventional face-to-face supervision. Therefore, mobile applications and telemedicine platforms should be viewed as potential delivery and adherence-support tools, and their incremental benefit over standard exercise guidance still requires further evaluation.

The COVID-19 pandemic accelerated the use of telemedicine in nephrology and dialysis care, especially for remote follow-up, patient education, and continuity of care when in-person visits were restricted. In some settings, telehealth visits were used to replace or supplement routine clinical encounters for dialysis patients, and patient perspectives from in-center hemodialysis suggested that telemedicine may be acceptable when incorporated appropriately into dialysis workflows. These experiences indicate that telemedicine may help maintain continuity of care, reduce unnecessary travel, and strengthen communication between patients and providers (39). However, the long-term effectiveness of telemedicine for sarcopenia-specific outcomes, such as handgrip strength, gait speed, skeletal muscle mass, exercise adherence, and hospitalization, remains insufficiently established.

From a global perspective, the implementation of mobile health applications and telemedicine platforms must also consider differences in digital infrastructure, socioeconomic status, language, culture, and health literacy. In low- and middle-income countries, limited internet access, unstable digital infrastructure, lack of smartphones, and insufficient reimbursement mechanisms may restrict the use of digital health tools. Among older MHD patients, visual impairment, cognitive decline, low digital literacy, and fear of technology may further reduce acceptance and adherence (40). Therefore, mobile applications and telemedicine platforms should be designed with simple interfaces, multilingual content, culturally adapted education materials, caregiver involvement, and offline or low-bandwidth functions when necessary.

Overall, mobile applications and telemedicine platforms are among the more clinically feasible digital health tools for supporting sarcopenia management in MHD patients. They may improve self-management, exercise adherence, dietary recording, remote follow-up, and continuity of care. However, current evidence remains heterogeneous, and many studies focus on general chronic disease management rather than MHD-specific sarcopenia outcomes. Future research should include internationally representative populations and compare app-based, telemedicine-based, and conventional management approaches using clinically meaningful outcomes, such as muscle strength, muscle mass, physical performance, quality of life, hospitalization, and long-term adherence.

### Virtual reality and augmented reality

Virtual reality (VR) and augmented reality (AR) are emerging digital technologies that may support rehabilitation training by creating immersive, interactive, and gamified exercise environments. VR refers to a computer-generated environment that allows users to interact with simulated scenarios, whereas AR overlays virtual information onto the real-world environment. In the context of MHD, these technologies may provide structured rehabilitation guidance, enhance exercise engagement, and reduce the monotony of conventional exercise programs. However, current evidence for VR/AR in MHD-associated sarcopenia is preliminary and mainly based on feasibility or qualitative studies. Therefore, VR and AR should currently be regarded as early-stage or validation-stage interventions rather than clinically established rehabilitation tools for MHD-associated sarcopenia. Hu et al. (41) explored the experiences of MHD patients participating in VR-based rehabilitation training and found that the VR system may enhance exercise motivation, improve exercise experience, and alleviate negative emotions. These findings suggest that VR may have value in improving patient participation and adherence, particularly among patients who find conventional exercise training repetitive or difficult to maintain. However, because this evidence mainly reflects patient experience and perceived benefits, it cannot confirm definitive effects on objective functional outcomes, such as handgrip strength, gait speed, skeletal muscle mass index, 6-min walk distance, hospitalization, or mortality. Therefore, VR/AR should be described as a potentially useful strategy for improving engagement and exercise experience, while its effects on physical function and sarcopenia-related clinical outcomes still require confirmation through randomized controlled trials.

The clinical application of VR/AR in MHD patients also requires careful safety evaluation. During intradialytic exercise, patients may experience blood pressure instability, intradialytic hypotension, muscle cramps, fatigue, and post-dialysis weakness (42). In addition, vascular access protection is a major concern during rehabilitation training, especially when upper-limb movement or immersive interactive tasks are involved. Older MHD patients may also be more vulnerable to dizziness, visual fatigue, motion sickness, impaired balance, and fall risk when using immersive VR systems (43). Therefore, VR/AR-based rehabilitation should be introduced cautiously, with appropriate supervision by healthcare professionals, especially during early use, intradialytic sessions, or in patients with frailty, visual impairment, cardiovascular instability, or poor balance.

In addition to safety concerns, several practical barriers may limit the broader implementation of VR/AR in MHD-associated sarcopenia management (44). These include equipment cost, device maintenance, infection control in dialysis units, technical complexity, staff training requirements, and patient acceptance. Whether VR/AR systems are suitable for home-based rehabilitation also remains uncertain. Home use may improve accessibility and continuity, but it may also increase risks related to unsupervised exercise, falls, improper device use, and poor adherence. Therefore, future studies should evaluate not only the effectiveness of VR/AR interventions, but also their feasibility, safety, usability, cost-effectiveness, and suitability for both dialysis-unit and home-based settings.

Overall, VR and AR technologies may provide an innovative approach to improving rehabilitation engagement and exercise experience in MHD patients. However, the current evidence remains insufficient to conclude that these technologies effectively improve sarcopenia-related functional outcomes. Future randomized controlled trials with adequate sample sizes and long-term follow-up are needed to compare VR/AR-based rehabilitation with conventional resistance exercise, aerobic exercise, therapist-supervised rehabilitation, and tele-rehabilitation. Clinically meaningful outcomes should include handgrip strength, gait speed, skeletal muscle mass index, 6-min walk distance, exercise adherence, quality of life, adverse events, hospitalization, and patient acceptability. Until such evidence is available, VR/AR should be presented as a promising but still investigational modality for MHD-associated sarcopenia management.

## Effects of digital health technologies in the management of sarcopenia in patients undergoing maintenance hemodialysis

### Improving the efficiency of sarcopenia identification and diagnosis

By integrating multiple data sources with intelligent algorithms, digital health technologies may facilitate earlier and more standardized identification of sarcopenia risk in patients undergoing MHD. Digital platforms can incorporate validated screening instruments, wearable-derived activity indicators, muscle function assessments, dialysis-related parameters, and biochemical data to support risk stratification. However, their role should currently be understood as supportive rather than diagnostic because sarcopenia diagnosis still requires standardized assessment of muscle strength, muscle mass, and physical performance. Wang et al. (45) compared the performance of five sarcopenia screening tools in MHD patients and found that the SARC-CalF questionnaire demonstrated the highest diagnostic accuracy. Digital health platforms may incorporate such screening instruments into mobile applications or electronic follow-up systems, thereby improving screening efficiency and expanding coverage. For example, regular app-based or platform-based screening may help identify patients who require further assessment of handgrip strength, gait speed, skeletal muscle mass, or nutritional status. Nevertheless, digital screening should not replace clinical evaluation, especially in MHD patients whose body composition and physical performance may be influenced by fluid overload, ultrafiltration, fatigue, and dialysis timing.

Machine learning may provide additional support for early risk identification. Tukhtaev et al. (28) used machine learning models to predict sarcopenia and highlighted the importance of feature selection in improving model performance and interpretability. Such approaches may be useful for processing multidimensional data, such as age, body composition, activity patterns, nutritional indicators, inflammatory markers, and functional measurements. However, many machine-learning models have not been specifically validated in MHD populations. Therefore, their findings should be interpreted cautiously, and future studies should evaluate whether these models remain accurate when dialysis-specific variables, such as dialysis vintage, ultrafiltration volume, interdialytic weight gain, dialysis adequacy, and uremic toxin burden, are included.

Musculoskeletal ultrasound may also contribute to sarcopenia assessment in MHD patients. Tao et al. (46) investigated the value of musculoskeletal ultrasound in the assessment of sarcopenia in MHD patients and found that gastrocnemius muscle thickness was positively correlated with handgrip strength and skeletal muscle mass index. The authors also proposed ultrasound-based diagnostic cutoff values for sarcopenia. In the future, integration of high-resolution musculoskeletal ultrasound with AI-assisted image analysis may help standardize muscle assessment and reduce operator dependence. However, AI-assisted ultrasound remains a validation-stage application. Multicenter studies are needed to determine whether it improves diagnostic accuracy, clinical workflow, and prediction of sarcopenia-related outcomes compared with conventional assessment methods.

Overall, digital technologies may improve the efficiency and coverage of sarcopenia screening in MHD patients, but current evidence is stronger for screening support than for definitive diagnosis. Future diagnostic studies should compare digital tools with established diagnostic criteria and evaluate sensitivity, specificity, calibration, and clinical utility in dialysis-specific populations.

### Optimizing the effects of exercise and nutritional interventions

Digital health technologies may support exercise and nutritional interventions in patients undergoing MHD by improving intervention delivery, adherence monitoring, real-time feedback, individualized adjustment, and long-term maintenance. However, it is important to distinguish two different types of evidence. The first concerns the effectiveness of exercise or nutritional interventions themselves, such as resistance training, intradialytic exercise, multicomponent exercise, and dietary management. The second concerns whether digital tools provide additional benefits by improving the accessibility, adherence, personalization, and sustainability of these interventions. At present, evidence supporting exercise and nutritional interventions in MHD-associated sarcopenia is relatively stronger than evidence demonstrating the independent or incremental effectiveness of digital delivery.

Exercise interventions are clinically important for improving sarcopenia-related outcomes in MHD patients. Wang et al. (47) reported that cognitive-motor dual-task guided therapy significantly improved handgrip strength, skeletal muscle mass, walking speed, activities of daily living, and balance and mobility in MHD patients with sarcopenic obesity. Li et al. (32) confirmed that resistance exercise significantly improved handgrip strength, 6-min walk distance, muscle mass, and serum albumin levels in MHD patients. Li et al. (31) further compared different exercise training methods for MHD patients with sarcopenia and highlighted the value of structured exercise intervention. These findings support the clinical importance of exercise interventions, while digital platforms may serve as delivery and monitoring tools. However, the incremental benefit of digital delivery over conventional supervised exercise remains insufficiently quantified.

Digital tools may facilitate the implementation of exercise programs by providing exercise videos, remote supervision, wearable-based activity monitoring, symptom check-ins, progress feedback, and reminders (48). For example, wearable devices can record step count, sedentary time, activity intensity, and exercise completion, while mobile applications and telemedicine platforms can help healthcare professionals adjust exercise prescriptions according to patients’ fatigue, dialysis schedule, blood pressure changes, and self-reported symptoms. These functions may be particularly useful in MHD patients because exercise tolerance may vary between dialysis and non-dialysis days. Nevertheless, current evidence does not yet clearly establish whether digitally supported exercise programs are superior to face-to-face supervised exercise or standard rehabilitation guidance. Chen et al. (33) developed and implemented an intradialytic exercise intervention program for patients with MHD-associated sarcopenia and found that the intervention group showed a lower prevalence of sarcopenia and better outcomes in handgrip strength, gait speed, and calf circumference than the control group. This study provides evidence for the effectiveness of structured intradialytic exercise, but it should not be interpreted as direct evidence for the effectiveness of digital health technologies. Rather, digital platforms may provide a practical pathway for scaling similar exercise programs through standardized exercise instruction, adherence tracking, safety alerts, remote professional supervision, and longitudinal outcome monitoring. Future studies should directly compare conventional intradialytic exercise, digitally supported intradialytic exercise, home-based tele-exercise, and usual care to determine the added value of digital delivery.

VR-based rehabilitation represents another potential digital approach to exercise engagement. Hu et al. (41) showed that VR-based rehabilitation training may improve exercise motivation and exercise experience in MHD patients. These findings suggest that immersive and gamified digital tools may help reduce the monotony of conventional exercise and improve participation. However, because the current evidence is mainly qualitative or exploratory, VR-based rehabilitation should be interpreted as a promising strategy for enhancing engagement rather than as a clinically validated intervention for improving sarcopenia-related functional outcomes. Its effects on handgrip strength, gait speed, skeletal muscle mass index, 6-min walk distance, adverse events, and long-term adherence still require confirmation through randomized controlled trials.

Nutritional intervention is another essential component of sarcopenia management in MHD patients. Protein-energy wasting, dialysis-related amino acid loss, inflammation, metabolic acidosis, and uremic toxin accumulation may all contribute to muscle wasting. Digital tools may support nutritional management through dietary recording, protein intake tracking, fluid intake monitoring, app-based education, remote dietitian consultation, and integration of dietary information with laboratory indicators (49). These laboratory indicators may include serum albumin, phosphorus, blood urea nitrogen, creatinine, C-reactive protein, and other markers related to nutrition, inflammation, and uremic toxin burden. However, mobile applications and digital dietary records should be regarded as supportive tools and cannot replace individualized assessment by nephrologists, nurses, or renal dietitians. Martino et al. (50) reported that, among older adults with chronic kidney disease receiving conservative treatment, a low-protein diet could confer significant benefits without worsening nutritional status. Although this study was not conducted in MHD patients with sarcopenia, it highlights the importance of individualized nutritional strategies in kidney disease populations. For MHD patients, nutritional management is more complex because adequate protein intake must be balanced with phosphorus control, fluid restriction, dialysis adequacy, and inflammation-related catabolism. Therefore, digital nutrition tools should be designed to support personalized dietary monitoring and professional decision-making rather than provide uniform dietary recommendations.

The conceptual continuous protein sensor proposed by Gottlieb et al. (20) may offer a future direction for real-time nutritional and metabolic monitoring. However, this technology remains a proof-of-concept tool and has not been clinically validated for MHD-associated sarcopenia. It should therefore be discussed as a future research direction rather than as a currently available clinical tool. At present, more feasible digital approaches include dietary logging applications, remote nutrition counseling, patient education platforms, and integration of dietary records with dialysis-related and biochemical data. Xia and Wang (51), in a quasi-randomized controlled trial, found that the teach-back strategy significantly improved hemodialysis-related knowledge, self-efficacy, and self-management ability in MHD patients. This finding supports the value of structured education in improving self-management. Digital platforms may extend teach-back-based education by providing interactive learning modules, reminders, repeated knowledge review, feedback, and remote communication with healthcare professionals. However, evidence is still needed to determine whether digital teach-back interventions can improve sarcopenia-specific outcomes, such as exercise adherence, dietary quality, muscle strength, physical performance, and long-term maintenance.

Overall, exercise and nutritional interventions are important components of sarcopenia management in MHD patients, and digital health technologies may enhance their delivery, monitoring, personalization, and sustainability. However, the current evidence more strongly supports the effectiveness of exercise and nutritional strategies themselves than the independent added value of digital delivery. Future studies should quantify whether digital platforms, wearable monitoring, tele-rehabilitation, app-based nutrition management, or VR-based exercise provide additional benefits over conventional supervised interventions in terms of muscle strength, muscle mass, physical performance, adherence, safety, cost-effectiveness, and quality of life.

### Improving quality of life and psychological wellbeing

Sarcopenia affects not only physical function in patients undergoing MHD but also psychological wellbeing and overall quality of life (52, 53). Reduced muscle strength, limited mobility, fatigue, dependence on others, and fear of functional decline may increase anxiety, depressive symptoms, and perceived illness burden in this population. Digital health technologies may contribute to psychological support by improving access to health information, strengthening communication with healthcare professionals, supporting remote follow-up, enhancing self-management confidence, and providing more engaging rehabilitation experiences. However, the current evidence linking digital health technologies directly to improved psychological outcomes in MHD-associated sarcopenia remains limited and should be interpreted cautiously.

Different digital or digitally supported interventions have different levels of evidence for psychological and quality-of-life outcomes. VR-based rehabilitation may contribute to improved exercise experience, motivation, and emotional engagement. Hu et al. (41) found that MHD patients participating in VR-based rehabilitation training reported enhanced exercise motivation, improved physical experience, and alleviation of negative emotions. These findings suggest that immersive and gamified rehabilitation may help reduce boredom and improve patients’ willingness to participate in exercise. Nevertheless, this evidence remains preliminary and is mainly based on patient experience rather than robust clinical outcomes. Therefore, VR-based rehabilitation should be described as a potential strategy that may contribute to psychological wellbeing and exercise engagement, while its sustained effects on quality of life, anxiety, depression, and sarcopenia-related functional outcomes still require confirmation through randomized controlled trials.

Structured rehabilitation programs may also improve quality of life by enhancing physical function. Wang et al. (47) reported that cognitive-motor dual-task guided therapy improved quality-of-life scores in MHD patients with sarcopenic obesity. However, this intervention should not be interpreted simply as a digital health tool unless it is delivered or supported through digital platforms. Its findings mainly indicate that structured rehabilitation and functional training may improve quality of life, whereas digital technologies may serve as potential delivery, monitoring, or feedback tools. Therefore, the psychological and quality-of-life benefits observed in such rehabilitation studies should not be directly attributed to digital health technologies without evidence comparing digitally supported and non-digital delivery formats.

Mobile applications, telemedicine platforms, and remote follow-up systems may contribute to psychological wellbeing through different mechanisms. These tools can provide regular symptom reporting, exercise reminders, medication and dietary guidance, remote consultation, peer support, and timely feedback from healthcare professionals (54). For MHD patients with sarcopenia, such functions may help reduce uncertainty, improve perceived support, and strengthen self-efficacy in long-term disease management. Patient portals and remote support platforms may also improve communication between patients and healthcare teams, which may be particularly important for older patients, patients with mobility limitations, or those who experience post-dialysis fatigue. However, current evidence remains insufficient to determine whether these tools directly improve depression, anxiety, or quality-of-life outcomes in MHD-associated sarcopenia.

Digital health literacy is another important factor influencing the psychological and behavioral effects of digital interventions. International studies indicate that digital health literacy, technology confidence, and access to reliable digital resources affect technology adoption and self-management among older adults (55–57). For MHD patients, adequate digital health literacy may improve the ability to access reliable health information, use mobile applications, communicate through telemedicine platforms, and interpret digital feedback on exercise, diet, and symptoms. However, digital health literacy should be regarded as a facilitator of technology use rather than direct evidence that digital technologies improve sarcopenia outcomes. Low digital literacy may increase frustration, reduce adherence, and widen health inequalities, especially among older adults, patients with lower educational attainment, and those with limited access to digital infrastructure.

From a global perspective, the psychosocial effects of digital health technologies may vary across healthcare systems, cultures, socioeconomic contexts, and levels of digital infrastructure (55, 58, 59). In high-resource settings, patient portals, remote follow-up systems, and telemedicine platforms may improve continuity of care and perceived support. In low- and middle-income settings, limited internet access, device cost, language barriers, low digital literacy, and insufficient reimbursement may reduce the benefits of digital interventions or increase the burden of use. Future digital health interventions for MHD-associated sarcopenia should therefore include culturally adapted education, simple interfaces, caregiver involvement, accessible language, and low-bandwidth or offline functions when appropriate.

Overall, digital health technologies may contribute to improved quality of life and psychological wellbeing in MHD patients with sarcopenia by enhancing engagement, self-efficacy, communication, remote support, and perceived control over long-term management. However, the strength of evidence differs across modalities. VR-based rehabilitation may improve exercise experience and negative emotions, but the evidence remains preliminary. Mobile applications and telemedicine platforms may enhance social support and self-management confidence, but their effects on psychological outcomes require further validation. Digital health literacy may facilitate technology adoption and health behavior change, but it should not be equated with direct improvement in sarcopenia outcomes. Future studies should use validated quality-of-life scales, anxiety and depression measures, adherence indicators, and long-term follow-up to clarify the psychosocial benefits of digital health technologies in MHD-associated sarcopenia.

## Challenges and recommendations

### Technological maturity, clinical validation, and interoperability

Many digital health technologies remain at different stages of development, and their long-term effectiveness, safety, cost-effectiveness, and clinical applicability in the management of sarcopenia among patients undergoing MHD have not yet been fully established (60, 61). Therefore, it is necessary to distinguish between technologies that are already clinically feasible, technologies that are undergoing clinical validation, and conceptual or proof-of-concept technologies. Without this distinction, preliminary or experimental tools may be overinterpreted as mature clinical solutions.

First, some digital tools are already clinically available and can be used to support monitoring, communication, and follow-up. These include wearable activity monitors, pedometers, smart watches, mobile health applications, telemedicine platforms, remote follow-up systems, electronic body-weight records, and blood pressure monitoring systems. In MHD patients, these tools may help record physical activity, sedentary time, exercise adherence, body weight, blood pressure, interdialytic weight gain, dietary intake, and patient-reported symptoms. They are therefore useful for supportive management and continuity of care. However, their direct effectiveness on MHD-specific sarcopenia outcomes, such as skeletal muscle mass, handgrip strength, gait speed, 6-min walk distance, hospitalization, quality of life, and mortality, remains insufficiently quantified. Future studies should determine whether these clinically available tools provide additional benefit beyond conventional face-to-face assessment and follow-up.

Second, several technologies have substantial research potential but still require clinical validation in MHD populations. These include AI-based sarcopenia prediction models, machine-learning-assisted gait analysis, AI-assisted musculoskeletal ultrasound, and VR/AR-based rehabilitation (62, 63). Such tools may support risk stratification, automated assessment, personalized intervention, and rehabilitation engagement. However, many existing models or interventions have been developed in general older adults, chronic disease populations, or pilot rehabilitation settings rather than in MHD patients with sarcopenia. Their diagnostic accuracy, predictive performance, safety, and clinical usefulness, therefore, cannot be assumed. Before routine clinical adoption, these tools require external validation, calibration across different dialysis populations, transparent reporting of algorithmic performance, comparison with standard clinical assessments, and evaluation of their effects on clinically meaningful outcomes.

Third, some technologies should be regarded as conceptual or proof-of-concept tools rather than currently available clinical applications. For example, the continuous protein sensor proposed by Gottlieb et al. (20) is still in the conceptual validation stage and has not been clinically validated for MHD-associated sarcopenia. Similarly, potential uremic-toxin biosensors, real-time amino acid loss monitoring systems, and experimental metabolic biosensors may offer future opportunities for precision monitoring of nutrition, metabolism, and toxin burden, but they remain technically and clinically immature (64). These tools should therefore be presented as future research directions rather than as established components of sarcopenia management in MHD patients.

In addition to technological maturity, interoperability remains a major barrier to clinical implementation. Poor interoperability across wearable devices, mobile applications, telemedicine systems, dialysis information systems, laboratory databases, and electronic health records can result in fragmented data environments (17, 61, 65). For MHD-associated sarcopenia, this fragmentation is particularly problematic because meaningful assessment requires integration of physical activity, muscle strength, nutritional intake, dialysis adequacy, ultrafiltration volume, interdialytic weight gain, inflammatory markers, and laboratory indicators related to uremic toxin burden. If these data remain isolated across platforms, clinicians may be unable to generate comprehensive risk assessments or timely intervention adjustments.

To address these challenges, future digital health systems for MHD-associated sarcopenia should be developed according to standardized data formats, interoperable technical frameworks, and clinically meaningful reporting structures. Digital platforms should allow secure integration of wearable-derived activity data, patient-reported outcomes, dialysis machine data, laboratory results, and electronic health records. At the same time, validation studies should evaluate not only technical performance but also clinical effectiveness, usability, workflow integration, safety, equity, and cost-effectiveness. Such efforts would help promote the transition of digital health technologies from technical feasibility to clinical validation and, ultimately, to patient-centered implementation in MHD-associated sarcopenia management.

### Differences in digital literacy and technology acceptance

Patients undergoing MHD are often older adults and may have limited digital literacy, which can create barriers to the use of digital health technologies and contribute to reluctance toward technology adoption (55). Digital health literacy is not only related to the ability to operate smartphones, wearable devices, or telemedicine platforms, but also to the ability to understand digital feedback, judge the reliability of online health information, protect personal privacy, and communicate effectively with healthcare professionals through digital platforms. Therefore, differences in digital literacy may directly influence the accessibility, adherence, and effectiveness of digital health interventions in MHD-associated sarcopenia.

Existing international evidence suggests that digital health technology use is unevenly distributed across age, education, income, ethnicity, and socioeconomic status. Tuitert et al. (55) showed that the use of digital health technologies varies across socioeconomic groups, with lower uptake among older individuals and those with lower educational attainment. Ferreira-Brito et al. (66) further found that non-clinical factors, such as computer confidence and self-efficacy, negatively influenced the user experience and feasibility of digital health technologies among older adults. These findings indicate that digital health interventions may unintentionally widen health inequalities if they are designed without considering the needs of older adults and socially disadvantaged groups. Rousseau et al. (67) also highlighted that digital health literacy assessment may reveal disparities among diverse older adults, suggesting that digital health implementation should be accompanied by equity-oriented evaluation and support.

However, the barriers to technology acceptance are not limited to individual-level digital literacy. From a global perspective, implementation of digital health technologies in MHD populations may be affected by language differences, cultural adaptation, digital infrastructure, dialysis service models, reimbursement systems, and access to smart devices (56, 57). In high-income countries, patient portals, remote monitoring systems, and telemedicine platforms may be more easily integrated into existing electronic health records and dialysis care pathways. In contrast, in low- and middle-income countries, unstable internet access, limited availability of smartphones, insufficient digital infrastructure, shortage of trained healthcare personnel, and lack of reimbursement mechanisms may restrict the use of digital tools.

Differences in dialysis service models may also affect the feasibility of digital health interventions (58). For example, in-center hemodialysis, home hemodialysis, and peritoneal dialysis are organized differently across countries and healthcare systems. Digital tools that are feasible in a highly integrated dialysis network may not be directly applicable in settings where dialysis services are fragmented or where electronic health records are not interoperable. Similarly, reimbursement policies may determine whether telemedicine consultations, remote exercise supervision, nutritional counseling, or wearable monitoring can be sustainably implemented. Therefore, technology acceptance should not be understood only as a patient-level issue, but also as a system-level issue involving healthcare infrastructure, payment policy, workforce capacity, and clinical workflow.

Language and cultural differences should also be considered (59). Health education materials, app interfaces, symptom questionnaires, dietary guidance, and exercise instructions need to be culturally and linguistically adapted. For MHD patients with sarcopenia, dietary recommendations are especially context-specific because protein intake, phosphorus control, sodium restriction, and fluid management may differ according to local dietary habits and healthcare resources. If digital platforms provide standardized content without cultural adaptation, patients may find the recommendations difficult to understand or implement. Therefore, future digital health tools should include multilingual interfaces, culturally appropriate educational materials, and flexible dietary and exercise modules.

Older MHD patients may face additional barriers related to physical and cognitive function. Visual impairment, hearing loss, hand weakness, cognitive decline, fatigue after dialysis, and fear of technology may reduce their ability to use mobile applications, wearable devices, or telemedicine platforms independently (68, 69). Caregiver involvement may therefore be important, especially for patients with frailty or severe sarcopenia. Digital tools should adopt user-centered design principles, such as large fonts, simple navigation, voice prompts, low-burden data entry, offline functions, and caregiver-accessible interfaces. Such design strategies may help improve usability and reduce the burden of digital participation.

Future implementation should therefore combine digital health literacy training with accessible technology design and system-level support. Multimedia education, face-to-face demonstrations, repeated practice, caregiver participation, and technical assistance may help older MHD patients develop confidence in using digital tools. At the same time, healthcare professionals should avoid assuming that all patients can benefit equally from digital interventions. Instead, digital readiness should be assessed before implementation, and alternative non-digital options should remain available for patients who are unable or unwilling to use digital technologies. This approach may help ensure that digital health technologies improve, rather than exacerbate, equity in sarcopenia management among MHD patients.

### Data privacy, security, and ethical challenges

Digital health technologies face substantial privacy, security, and ethical challenges when collecting, storing, transmitting, and analyzing large volumes of sensitive health data (65). In the management of sarcopenia among patients undergoing MHD, digital platforms may involve multiple types of personal and clinical information, such as wearable-derived activity data, body weight, blood pressure, dietary intake, dialysis adequacy, laboratory indicators, patient-reported symptoms, and rehabilitation records. These data are highly sensitive because they may reflect not only patients’ disease status, but also their daily behavior, functional capacity, treatment adherence, and prognosis. Data breaches, misuse, unauthorized access, or inappropriate secondary use may therefore cause serious harm to patients.

The ethical risks of digital health technologies are particularly important when artificial intelligence is involved. The “black-box” nature of some AI algorithms may make it difficult for clinicians and patients to understand how risk predictions or intervention recommendations are generated (70). In addition, algorithmic bias may occur if AI models are developed using data from limited populations or specific regional healthcare systems. Such models may perform poorly in older adults, patients with low socioeconomic status, ethnic minority groups, or patients from countries with different dialysis practices and healthcare infrastructures. Therefore, AI-based sarcopenia prediction or decision-support tools should undergo external validation, fairness evaluation, and transparent reporting before being used in routine clinical decision-making.

Another ethical concern is the boundary between supportive digital tools and clinical decision-making. Clinically available tools, such as wearable devices, mobile applications, and telemedicine platforms, may support monitoring and communication, but they should not replace professional assessment by nephrologists, nurses, rehabilitation specialists, or dietitians. Technologies that remain under validation, such as AI-based prediction models or VR/AR rehabilitation systems, should be used cautiously and with a clear explanation of their limitations. Conceptual or proof-of-concept technologies, such as continuous protein sensors or potential uremic-toxin biosensors, should not be incorporated into clinical decision-making until their technical reliability, clinical validity, safety, and cost-effectiveness have been established.

Data governance also needs to be considered in an international context. Regulations for digital health data protection, patient consent, data sharing, cloud storage, and cross-border data transfer differ across countries and regions. These differences may affect the global implementation of digital health platforms for MHD-associated sarcopenia. Future studies and implementation programs should ensure that digital tools comply with local legal and regulatory requirements while following internationally accepted principles of transparency, accountability, security, and patient autonomy (61, 70).

Moving forward, stronger legal, ethical, and technical safeguards should be established for the application of digital health technologies in MHD-associated sarcopenia. Clear rules are needed regarding data ownership, patient consent, access authority, storage duration, data sharing, and responsibility for AI-assisted recommendations. Technical measures, such as data encryption, identity authentication, access control, audit trails, secure cloud storage, and de-identification procedures, should be implemented to reduce the risk of data leakage and misuse. At the same time, patients should be informed in understandable language about what data are collected, how the data are used, who can access them, and whether algorithmic tools are involved in decision support. These safeguards are necessary to ensure that digital health technologies are applied in a fair, transparent, secure, and patient-centered manner.

### Need for MHD-specific digital monitoring frameworks

Most existing studies on digital health technologies for sarcopenia management have focused on general older adults or broad chronic disease populations, without adequately accounting for the distinctive features of MHD, such as volume fluctuations, dialysis-cycle-related activity patterns, and uremic effects on muscle metabolism (4, 71). These factors are central to the development, assessment, and management of sarcopenia in MHD patients. Future digital health interventions should not simply transplant models from non-dialysis populations, but should develop MHD-specific digital monitoring frameworks. To clarify the relationship between dialysis-specific determinants and digital monitoring pathways, the major MHD-specific factors relevant to sarcopenia are summarized in Table 3.

**Table 3:** Dialysis-specific determinants of sarcopenia and potential digital monitoring pathways in MHD patients.

MHD-specific determinant	Relevance to sarcopenia	Possible indicators	Potential digital integration pathway	Key limitations
Dialysis adequacy	Inadequate dialysis may contribute to the accumulation of uremic solutes, chronic fatigue, poor appetite, inflammation, and catabolic status, thereby aggravating muscle wasting and functional decline.	Kt/V, urea reduction ratio, blood urea nitrogen, dialysis duration, treatment frequency, residual kidney function	Digital platforms may integrate dialysis adequacy indicators from dialysis records or electronic health systems with fatigue scores, dietary intake, physical activity, handgrip strength, and gait speed to identify patients at higher risk of functional decline.	Dialysis adequacy alone cannot fully explain sarcopenia risk; values may vary across dialysis prescriptions, residual kidney function, and patient adherence.
Uremic toxin burden	Uremic toxins may impair muscle metabolism, mitochondrial function, appetite, and inflammatory pathways, contributing to uremic sarcopenia and reduced exercise tolerance.	Blood urea nitrogen, creatinine, phosphorus, β2-microglobulin, indoxyl sulfate, p-cresyl sulfate	Digital tools cannot directly replace laboratory testing, but may integrate toxin-related laboratory indicators with Kt/V, dietary records, fatigue symptoms, physical activity decline, and muscle function measurements to generate risk alerts.	Real-time uremic toxin biosensors are not clinically mature; most toxin-related indicators still require laboratory testing and are not routinely measured in all centers.
Volume overload and interdialytic weight gain	Fluid overload may reduce exercise tolerance, worsen fatigue and dyspnea, and interfere with muscle mass or body composition assessment. Excessive interdialytic weight gain may also reflect poor fluid management and higher cardiovascular burden.	Pre- and post-dialysis body weight, interdialytic weight gain, ultrafiltration volume, blood pressure variability, edema, dyspnea, bioelectrical impedance analysis	Digital systems may combine electronic weight records, home blood pressure monitoring, bioelectrical impedance data, symptom reporting, and wearable-derived activity data to distinguish true functional decline from volume-related fluctuations.	Body weight and impedance-derived parameters may be affected by hydration status; measurement timing in relation to dialysis must be standardized.
Ultrafiltration burden and intradialytic instability	Rapid or excessive ultrafiltration may cause intradialytic hypotension, cramps, fatigue, and post-dialysis weakness, limiting exercise participation and rehabilitation tolerance.	Ultrafiltration volume, ultrafiltration rate, intradialytic blood pressure, hypotension episodes, cramps, post-dialysis fatigue	Dialysis machine data and blood pressure records can be integrated with exercise completion, fatigue scores, and adverse symptom reporting to guide safer timing and intensity of exercise interventions.	Requires interoperability between dialysis machines, electronic health records, and digital rehabilitation platforms; causal relationships with sarcopenia outcomes require validation.
Chronic inflammation	Inflammation promotes muscle protein breakdown, anorexia, reduced physical performance, and poor response to nutritional or exercise interventions.	C-reactive protein, interleukin-6, tumor necrosis factor-α, albumin, neutrophil-to-lymphocyte ratio	Digital platforms may integrate inflammatory biomarkers with appetite, dietary intake, activity levels, fatigue, and muscle strength to identify patients who may require closer nutritional or medical evaluation.	Biomarkers are usually measured intermittently; inflammatory markers are non-specific and may be affected by infection, vascular access complications, and comorbidities.
Metabolic acidosis	Metabolic acidosis may accelerate muscle proteolysis, impair protein metabolism, and worsen muscle weakness in kidney failure.	Serum bicarbonate, blood pH, total CO₂, anion gap, dialysis prescription parameters	Laboratory data may be linked with dietary intake, protein catabolism indicators, muscle strength, and physical performance measures to support risk stratification and individualized management.	Digital devices cannot directly assess acidosis without laboratory testing; optimal thresholds for predicting sarcopenia progression in MHD require further study.
Protein-energy wasting	Protein-energy wasting directly contributes to loss of muscle mass, reduced muscle strength, impaired immunity, and poorer prognosis in MHD patients.	Serum albumin, prealbumin, normalized protein catabolic rate, dietary protein and energy intake, body weight change, appetite, Subjective Global Assessment	Mobile dietary records, nutrition apps, remote dietitian consultation, and laboratory data can be integrated to monitor nutritional risk and support individualized dietary intervention.	Dietary records depend on patient input and may be inaccurate; serum albumin is affected by inflammation and hydration status, not nutrition alone.
Dialysis-related amino acid loss	Amino acid loss during dialysis may aggravate negative nitrogen balance and muscle wasting, especially when dietary intake is inadequate.	Estimated amino acid loss, protein intake, serum albumin, prealbumin, normalized protein catabolic rate, appetite, dietary records	Digital nutrition platforms may combine dialysis schedule, dietary protein intake, and laboratory nutrition markers to support personalized protein supplementation and timing of nutrition intervention.	Direct measurement of amino acid loss is uncommon in routine practice; continuous amino acid monitoring remains experimental.
Dialysis-cycle-related activity variation	MHD patients may experience different activity levels, fatigue, sleep patterns, and exercise tolerance on dialysis and non-dialysis days, which may influence rehabilitation planning and adherence.	Step count, sedentary time, activity intensity, sleep duration, heart rate, fatigue scores, exercise completion, dialysis-day versus non-dialysis-day activity patterns	Wearable devices and mobile apps can compare activity and fatigue patterns across dialysis and non-dialysis days, helping clinicians individualize exercise timing, intensity, and recovery periods.	Wearable data may be affected by device adherence, sensor accuracy, and patient behavior; clinically meaningful thresholds remain unclear.
Measurement bias caused by hydration status	Fluid retention may distort body composition assessment, especially bioelectrical impedance analysis and weight-based indicators, leading to misclassification of muscle mass or sarcopenia severity.	Timing of measurement relative to dialysis, pre- and post-dialysis weight, bioelectrical impedance parameters, edema status, ultrasound muscle thickness	Digital platforms may record dialysis timing, ultrafiltration volume, hydration status, and measurement conditions to contextualize BIA, ultrasound, handgrip strength, and gait speed results.	Standardized protocols for timing and interpretation are needed; hydration correction methods are not yet uniformly established.

This framework highlights that MHD-associated sarcopenia cannot be adequately monitored by general activity indicators alone. Instead, digital health systems should integrate dialysis prescription data, volume-related indicators, laboratory biomarkers, nutritional records, wearable-derived activity patterns, and patient-reported symptoms. Such integration may help clinicians distinguish true sarcopenic progression from dialysis-related physiological fluctuations, identify patients at high risk of muscle wasting, and tailor exercise or nutritional interventions more safely. However, many of these pathways remain conceptual and require prospective validation before routine clinical implementation.

Among these factors, volume status deserves particular attention because fluid overload and rapid fluid removal may affect body weight, body composition assessment, exercise tolerance, and physical performance in MHD patients (12, 72). Future digital platforms should record the timing of sarcopenia-related measurements in relation to dialysis sessions and integrate pre- and post-dialysis weight, interdialytic weight gain, ultrafiltration volume, blood pressure variability, and bioelectrical impedance data when available. This approach may reduce misinterpretation of hydration-related changes as true sarcopenic progression.

Uremic-toxin-related risk monitoring should be interpreted cautiously. Most current digital devices cannot directly measure uremic toxins in real time, and digital platforms should not be described as substitutes for laboratory testing. Instead, they may integrate laboratory indicators related to dialysis adequacy, toxin burden, nutrition, and inflammation, such as blood urea nitrogen, creatinine, phosphorus, beta2-microglobulin, indoxyl sulfate, p-cresyl sulfate, serum albumin, C-reactive protein, and interleukin-6 (4, 8, 26, 73, 74). When combined with dialysis adequacy indicators, dietary intake, physical activity patterns, and muscle function assessments, these data may support risk stratification for sarcopenia progression.

Digital monitoring should also account for dialysis-cycle-related activity patterns. MHD patients may show different levels of physical activity, sedentary behavior, sleep quality, fatigue, and exercise tolerance on dialysis and non-dialysis days (75). Wearable devices and mobile applications can compare step count, activity intensity, sedentary time, sleep duration, fatigue scores, and exercise completion across different phases of the dialysis cycle. Such information may help clinicians determine appropriate exercise timing, adjust exercise intensity, and reduce adverse events related to post-dialysis fatigue or intradialytic instability.

Finally, MHD-specific digital frameworks should help contextualize hydration-related bias in sarcopenia assessment. Muscle mass assessment in MHD patients is easily affected by water retention, edema, and the timing of measurement in relation to dialysis sessions (76). Therefore, digital platforms should record whether body composition, bioelectrical impedance analysis, ultrasound measurement, handgrip strength, or gait speed is assessed before or after dialysis. Integrating measurement timing, ultrafiltration volume, body weight changes, and volume status may help clinicians interpret sarcopenia assessments more accurately.

Overall, the management of sarcopenia in MHD patients requires digital frameworks that reflect the unique physiological and treatment-related features of dialysis. Volume fluctuations, uremic toxin burden, dialysis-cycle-related activity patterns, nutritional impairment, inflammation, metabolic acidosis, protein-energy wasting, and hydration-related measurement bias should all be incorporated into future digital health systems. Large-scale, multicenter, and internationally representative studies are needed to develop and validate these MHD-specific frameworks and to determine their effectiveness, safety, cost-effectiveness, clinical usability, and impact on sarcopenia-related outcomes.

## Conclusion

Digital health technologies may provide supportive tools for addressing several limitations of traditional sarcopenia management in patients undergoing MHD, such as insufficient continuity of follow-up, limited individualization of interventions, delayed monitoring, and poor long-term adherence. Clinically available tools, such as wearable activity monitors, mobile health applications, telemedicine platforms, remote follow-up systems, and digital body-weight or blood pressure monitoring systems, may help support physical activity tracking, exercise supervision, dietary recording, symptom reporting, patient education, and communication between patients and healthcare professionals.

However, current evidence suggests potential benefits rather than definitive clinical effectiveness. The effects of digital health technologies on MHD-specific sarcopenia outcomes, such as skeletal muscle mass, handgrip strength, gait speed, 6-min walk distance, hospitalization, quality of life, and mortality, remain insufficiently quantified. In particular, although VR/AR-based rehabilitation may improve exercise engagement and exercise experience, its effects on objective functional outcomes still require confirmation through randomized controlled trials. Similarly, AI-based prediction models and AI-assisted assessment tools show potential for risk stratification and individualized management, but most have not yet been externally validated in MHD-associated sarcopenia populations.

More internationally representative studies are needed to determine whether current findings are generalizable across different healthcare systems, dialysis service models, socioeconomic backgrounds, cultural contexts, and levels of digital infrastructure. Many existing studies are derived from specific regional or clinical settings, which may limit the global applicability of their conclusions. Future research should therefore include multicenter and multinational populations and should pay particular attention to older adults, patients with low digital literacy, socially disadvantaged groups, and populations in low- and middle-income countries.

Clinically available tools should be clearly distinguished from technologies that remain under clinical validation or at the conceptual/proof-of-concept stage. Wearable devices, mobile applications, and telemedicine platforms are relatively feasible for supportive monitoring and follow-up, whereas AI-based sarcopenia prediction models, AI-assisted musculoskeletal ultrasound, and VR/AR rehabilitation require further validation. Continuous protein sensors, uremic-toxin-related biosensors, and other experimental metabolic monitoring technologies should be regarded as future research directions rather than mature clinical tools.

Future studies should develop MHD-specific digital monitoring frameworks that integrate physical activity data with dialysis-related and biochemical indicators, such as interdialytic weight gain, volume status, blood pressure variability, ultrafiltration volume, dialysis adequacy, nutritional markers, inflammatory biomarkers, and laboratory indicators associated with uremic toxin burden. In addition, the incremental effectiveness, safety, cost-effectiveness, usability, and patient acceptability of different digital modalities should be compared with conventional care. Interdisciplinary collaboration among nephrologists, rehabilitation specialists, nurses, dietitians, engineers, data scientists, and health policymakers will be essential to promote the transition of digital health technologies from technical feasibility to clinically validated, patient-centered, and globally applicable tools for sarcopenia management in MHD patients.
